# Advances in the understanding of delayed cerebral ischaemia after aneurysmal subarachnoid haemorrhage

**DOI:** 10.12688/f1000research.6635.1

**Published:** 2015-11-02

**Authors:** Liam Flynn, Peter Andrews

**Affiliations:** 1 Centre for Clinical Brain Sciences, University of Edinburgh, Edinburgh, UK

**Keywords:** Cerebral ischaemia, subarachnoid, haemorrhage

## Abstract

Delayed cerebral ischaemia has been described as the single most important cause of morbidity and mortality in patients who survive the initial aneurysmal subarachnoid haemorrhage. Our understanding of the pathophysiology of delayed cerebral ischaemia is meagre at best and the calcium channel blocker nimodipine remains the only intervention to consistently improve functional outcome after aneurysmal subarachnoid haemorrhage. There is substantial evidence to support cerebral vessel narrowing as a causative factor in delayed cerebral ischaemia, but contemporary research demonstrating improvements in vessel narrowing has failed to show improved functional outcomes. This has encouraged researchers to investigate other potential causes of delayed cerebral ischaemia, such as early brain injury, microthrombosis, and cortical spreading depolarisation. Adherence to a common definition of delayed cerebral ischaemia is needed in order to allow easier assessment of studies using multiple different terms. Furthermore, improved recognition of delayed cerebral ischaemia would not only allow for faster treatment but also better assessment of interventions. Finally, understanding nimodipine’s mechanism of action may allow us to develop similar agents with improved efficacy.

## Introduction

Aneurysmal subarachnoid haemorrhage (aSAH) has an incidence of 6–11 per 100,000 people per year and accounts for only 5% of all strokes ^1– 4^. Despite this, aSAH is the cause of one third of all stroke-related years of potential life lost before the age of 65 ^5^. Approximately 70% of all people with aSAH will either die or require help with activities of daily living at six months after the initial injury ^5^. The mean age of onset of aSAH is 55 years and, when combined with its poor morbidity and mortality, it causes an enormous socioeconomic burden ^6, 7^. The significant morbidity attached to aSAH can be attributed to rebleeding, delayed cerebral ischaemia (DCI), hydrocephalus, and other medical complications, despite successful treatment of the ruptured aneurysm. Of these complications, DCI is the most important cause of morbidity and mortality in patients who survive the ruptured aneurysm ^5, 8, 9^. Between days 3 and 10 after the initial aSAH, 30–40% of patients will suffer DCI and half of these will have a poor outcome ^5, 10, 11^.

Our understanding of DCI is meagre at best. Conventionally, DCI was thought of as a neurological deficit observed at least three days after aSAH with radiological confirmation of large vessel narrowing and was often termed “vasospasm”. However, more contemporary articles question whether the relationship between angiographic cerebral vessel narrowing and neurological outcome is associative rather than causative and have highlighted the possibility of a multifactorial aetiology ^12– 15^. One of the problems with the disease and research surrounding DCI is the terminology applied. Terms include DCI, delayed ischaemic neurological deficit (DIND), delayed neurological deficit, secondary cerebral ischaemia, and vasospasm. In 2010, a consensus statement was issued defining DCI as a focal neurological impairment or decrease of ≥2 points on the Glasgow Coma Scale which lasts for ≥1 hour, is not apparent immediately after aneurysm occlusion, and cannot be attributed to other causes by means of clinical assessment, blood tests, or imaging ^16^. The Neurocritical Care Society’s consensus definition was similar for DCI and also defined vasospasm as radiological evidence of cerebral vessel narrowing with corresponding neurology ^17^.

## Cerebral artery narrowing

Over six decades ago, cerebral vessel narrowing was demonstrated by angiography after aSAH ^18^. A decade later, a link was found between cerebral vessel narrowing and focal neurology ^19^. Then in the late 1970s, it appeared that vessel narrowing was not only localised to the vascular territory of the aneurysmal bleed but also proportional to blood load and occurred between days 3 and 12 after the aSAH ^20, 21^. More contemporary authors found the onset of vessel narrowing started on day 3, was maximal by days 6–10, and lasted for up to two weeks ^22– 24^. The density, duration and volume of subarachnoid blood are key predictors of vessel narrowing ^21, 25^. Narrowing of cerebral arteries may cause a reduction in cerebral blood flow distal to the constricted vessel and contribute to secondary ischaemia ^26^. The cause of vessel narrowing after aSAH is unclear but is thought to involve oxyhaemoglobin release, an inflammatory-mediated response, decreased nitric oxide levels, and an increased concentration of endothelin-1 (ET-1) ^14^.

## Oxyhaemoglobin

Oxyhaemaglobin induces cerebral artery vasoconstriction *in vitro* and *in vivo* in primates, which is not seen with methaemoglobin or bilirubin ^27– 29^. It is thought that oxyhaemoglobin decreases the production of prostacyclin and increases prostaglandin E2 in vessel walls, thereby causing vasoconstriction. It can also inhibit endothelial-dependent relaxation. The oxidation of oxyhaemoglobin to methaemoglobin, which occurs spontaneously, causes lipid peroxidation and vasoconstriction ^30^. It is plausible that oxyhaemoglobin causes vasoconstriction by some or all of these mechanisms but attempts at modulating them have not completely reversed vessel narrowing or, importantly, improved outcomes.

## Nitric oxide

Nitric oxide, which is responsible for the relaxation of vascular smooth muscle cells, appears to be depleted after aSAH. This may be due to a number of reasons, one of which is that nitric oxide is scavenged by haemoglobin, released during the breakdown of subarachnoid blood, due to nitric oxide’s high affinity for haemoglobin ^31, 32^. In addition to this, the production of nitric oxide may also be decreased due to the down-regulation of endothelial and neuronal nitric oxide synthase, which occurs in spastic arteries after aSAH ^33– 35^. Both of these mechanisms will lead to a decrease in the bioavailability of nitric oxide, which is then unable to counteract the effects of the vasoconstrictor ET-1 ^36^. Furthermore, exogenous donors of nitric oxide, such as sodium nitroprusside and nitroglycerin, although associated with systemic side effects, have been shown to ameliorate cerebral artery narrowing ^37, 38^. In addition to the hypotension seen with these exogenous donors, there is also a concern that exposing nitric oxide to oxyhaemoglobin and deoxyhaemoglobin will lead to the formation of methaemoglobin, S-nitrosohaemoglobin and ferrous-nitrosyl-haemoglobin ^33^. Interestingly, Kida *et al.* note in their comprehensive review that inhaled nitric oxide acts as a selective pulmonary vasodilator and avoids the hypotension seen with intravenous administration. Animal studies have demonstrated a reduction in ischaemia-reperfusion injuries after nitric oxide inhalation in extrapulmonary organs after cardiac injury. These have also been supported by proof-of-concept human trials ^39^. The research discussed is used to support post-cardiac arrest ischaemia but Garry *et al.* also encourage further investigation of nitric oxide as a treatment of secondary brain injury in their review with reference to aSAH ^40^.

## Endothelin

Endothelin is key to maintaining the vascular tone of blood vessels, with ET-1 being the most potent endogenous activator of vasoconstriction. The amount of ET-1 in serum and plasma increases within minutes after the aSAH and peaks around days 3–4, the time at which DCI starts to occur. There also appears to be an excessive release of ET-1 by astrocytes around the time of onset of ischaemic symptoms ^41, 42^. ET-1 concentrations appear consistently elevated in patients with DCI. However, there are conflicting reports of ET-1 concentrations within the normal range in patients with radiological evidence of cerebral artery narrowing who do not have DCI ^43– 45^. Authors have questioned whether increased ET-1 marks ischaemic damage rather than arterial vessel narrowing in DCI ^14^. Therefore, there are a number of different mechanisms that could be contributing to the arterial narrowing commonly seen after aSAH.

## Alpha calcitonin gene-related peptide

Alpha calcitonin gene-related peptide (CGRP) is an endogenous neuropeptide and a potent vasodilator. CGRP exhibits its vasodilating properties by two mechanisms: one is nitric oxide and endothelium-dependent and the other is cyclic adenosine monophosphate mediated and is endothelium-independent ^46^. Endogenous CGRP appears to be released, and is subsequently depleted, after aSAH to combat cerebral vasoconstriction which has led to the theory that exogenous CGRP may be beneficial in managing DCI ^47– 49^. Because CGRP can act independently of endothelial cells, which are morphologically damaged after aSAH, it may be successful in treating DCI. A number of animal studies and three human trials have investigated the effect of CGRP on cerebral arteries after aSAH. All animal studies appear to show either a reversal or improvement in cerebral artery narrowing ^46^. The largest human trial, the European CGRP in aSAH study, demonstrated little improvement in morbidity or mortality from intravenous administration but noted that systemic side effects, such as hypotension, were limiting and suggested that intrathecal administration may be more beneficial, as endogenous CGRP acts on the abluminal side of vessel walls ^50^. A trial investigating the effect of CGRP after intrathecal administration is still awaited.

## Radiological evidence

An often-cited argument against cerebral vasoconstriction being a causative factor of DCI is that, whilst up to 70% of patients demonstrate cerebral vessel narrowing on angiography, only 40% of these will manifest neurological deficits and only 30% develop DCI ^51– 54^. However, it must be acknowledged that even the consensus definition of DCI provided in the introduction has its limitations ^16^. Patients with poor grade aSAH (World Federation of Neurosurgical Societies Grades IV and V), the group of patients most likely to develop DCI, are often sedated and mechanically ventilated and are particularly difficult to assess clinically ^55^. Therefore, it is likely that we are under-diagnosing and under-treating DCI in this group of patients. Furthermore, it may be that the degree of large cerebral vessel narrowing does not correlate well with symptom severity ^26^.

**Figure 1.**
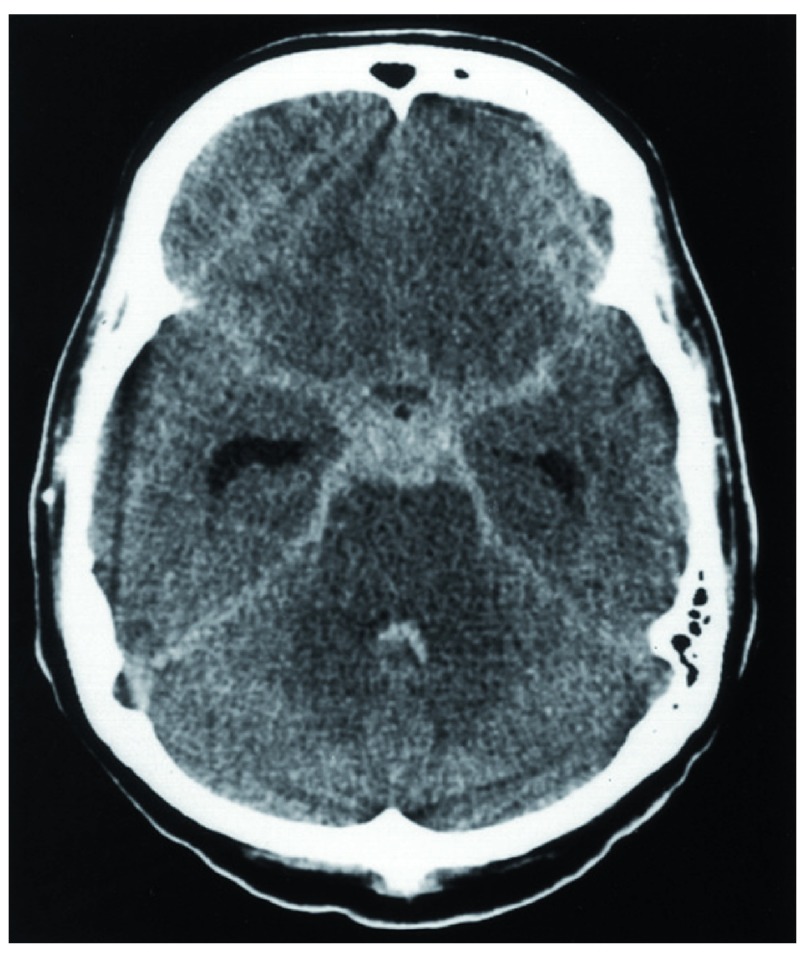
CT image of subarachnoid haemorrhage. Non-contrast CT scan of brain showing subarachnoid haemorrhage in classical “star sign” distribution with blood distributed along basal vessels.

Following a review of current tests available for the diagnosis of delayed cerebral ischaemia, Rodriguez *et al.* advise clinical examination and transcranial Doppler (TCD) in the screening and diagnosis of “vasospasm”. The authors reserve multi-modal magnetic resonance imaging (MRI) and computed tomography (CT) for specific situations, and acknowledge digital subtraction angiography (DSA) as the gold standard for diagnosis ( Figure 1) ^56^. Rabinstein *et al.* found that TCD and angiogram demonstrating cerebral vessel narrowing (termed vasospasm) only had a positive predictive value of 67% for cerebral infarction on CT ^8^. We would expect this to be higher if cerebral vessel narrowing was the primary cause of DCI. Rates of cerebral infarction in patients with evidence of cerebral vessel narrowing range between 24 and 35% using CT ^57, 58^, but have been found to be as high as 81% in some studies using MRI ^59^. In addition to this poor correlation between cerebral vessel narrowing and infarction, there is clinical evidence that up to 25% of delayed infarcts on CT are not in the same territory as the vessel narrowing, or are found in patients that did not demonstrate vessel narrowing at all ^60– 62^. Rabinstein *et al.* note that TCD and angiogram only agreed on the diagnosis of “vasospasm” in 73% of cases and so it could be that vessel narrowing simply wasn’t identified in patients who were later found to have evidence of infarcts on CT ^8^. Despite these conflicting messages, clinical studies do report that those patients with radiological evidence of cerebral vessel narrowing are at greater risk of DCI ^62, 63^.

Herz *et al.* directly visualised pial artery constriction after application of blood or microtrauma to pial arteries in animal studies ^64^. Further *in vitro* research has suggested that constriction of intraparenchymal arterioles occurs after aSAH and may contribute to DCI ^65^. Maximal luminal narrowing has been seen between days 3 and 7 and repeated *in vivo* in mouse studies. The correlation between decreased regional cerebral blood flow and microvascular constriction appears stronger than that seen with large vessel narrowing ^65– 67^. Uhl *et al.* identified constriction of small vessels in surgical patients within the first 72 hours after aSAH by spectral imaging, and Pennings *et al.* later directly observed cerebral arterioles contracting after aSAH ^68, 69^. Therefore, it may be that vessel narrowing is consistently occurring with DCI but that we are not visualising it because it is microvascular and not readily visible on catheter angiography or TCD ^56^.

CT perfusion scanning (CTP) may provide haemodynamic evidence to support the diagnosis of DCI. Dankbaar *et al.* evaluated the diagnostic value of CTP for DCI and reported 84% sensitivity, 79% specificity, and 88% positive predictive values ^70^. Sanelli *et al.* found that more CTP deficits occurred in patients with DCI than in those without ^71^. Dankbaar *et al.* later suggested that patients with DCI exhibit worse cerebral perfusion (measured on CTP) than patients without DCI even before focal signs occurred. Encouragingly, they demonstrated partial recovery in areas of poor perfusion, suggesting that DCI could be partly reversible ^72^. However, Killeen *et al.* concluded from their retrospective comparative study that CTP and DSA had similar test characteristics for identifying DCI in aSAH patients ^73^.

## Endothelin-antagonists

A shift in theory from cerebral vessel narrowing to a multifactorial aetiology occurred after the CONSCIOUS trials and a recent meta-analysis of pharmacological treatments for delayed cerebral ischaemia ^74– 76^. The meta-analysis demonstrated that, despite a reduction of cerebral vessel narrowing, no statistically significant effect on poor outcome was observed ^74^. However, the authors note that the dissociation between a reduction in cerebral vessel narrowing but not poor outcomes could result from methodological problems, sample size, and insensitivity of outcome measures, in addition to a multifactorial aetiology of DCI. The CONSCIOUS trials were multicentre randomised controlled trials (RCT) investigating the effect of clazosentan, an endothelin-A (ET-A) antagonist, on “vasospasm” after aSAH. The first of these trials, CONSCIOUS-1, demonstrated that, despite a significant reduction in angiographic cerebral vessel narrowing, there was little evidence to support its use to improve morbidity and mortality and it was associated with increased rates of pulmonary complications, hypotension and anaemia ^76^. CONSCIOUS-2 demonstrated no benefit from clazosentan in patients treated with surgical clipping, which led to the early termination of the trial ^75^. Laban *et al.* recently published a review of animal studies investigating endothelin receptor antagonists after experimental aSAH and found no improvement in functional outcomes ^77^. Perhaps more importantly, the review described insufficient animal data supporting endothelin receptor antagonists to warrant progression to a human trial. The authors also suggest that cerebral artery diameter, or “vasospasm”, is not a clinically relevant outcome measure in experimental aSAH studies ^77^.

**Figure 2.**
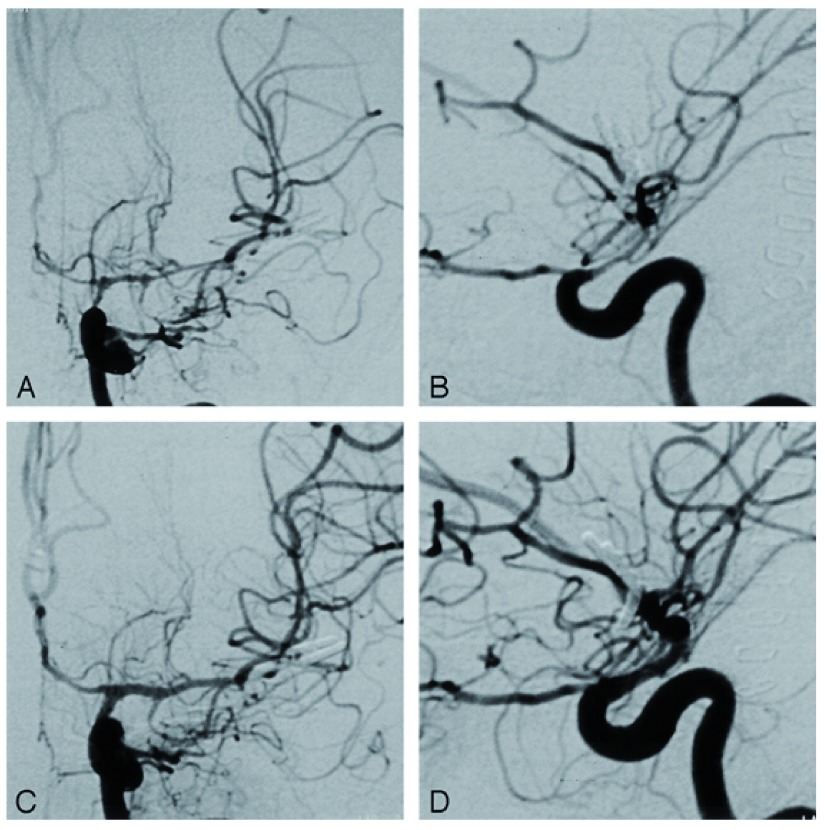
Angiograms demonstrating cerebral vessel narrowing after subarachnoid haemorrhage. **A** and **B**: Anteroposterior ( **A**) and lateral ( **B**) angiograms of the left internal carotid artery demonstrate vessel narrowing at the level of the carotid siphon, the terminal internal carotid artery, the A1 segment of the anterior cerebral artery and the middle cerebral artery. **C** and **D**: Anteroposterior ( **C**) and lateral ( **D**) angiograms obtained after intra-arterial injection of nimodipine.

The example of clazosentan appears to provide evidence that cerebral artery narrowing is not the sole cause of DCI. However, there is conflicting evidence as more invasive methods of reducing vessel narrowing can improve outcomes ( Figure 2). Kimball *et al.* reviewed 49 articles relating to interventional techniques to treat “vasospasm”. A total of 24 of the 27 publications (1,028 patients) reporting the use of transluminal balloon angioplasty noted an improvement in vessel diameter and neurological deficits. Twelve case series reported good angiographic and clinical results for patients who received papaverine (a vasodilator) administered approximate to the site of vessel narrowing ^78^. Both techniques were associated with significant side effects and the quality of the studies was reported as very low to moderate (based upon the GRADE classification system) ^79^. Nevertheless, the review does provide evidence that cerebral artery narrowing is likely to be strongly involved in the pathology of DCI.

## Nimodopine

The calcium channel antagonist nimodipine is the only proven intervention to reduce the incidence of DCI and improve outcomes after aSAH. Nimodopine was initially investigated as a vasodilator in the hope that it would aid post-ischaemic reperfusion, as it was thought that an increase in calcium in vascular smooth muscle cells led to “vasospasm” ^80, 81^. In 1989, the British Aneurysm Nimodipine Trial subsequently demonstrated a significant reduction in cerebral infarction rates and improved neurological outcomes at three months after aSAH ^82^. A Cochrane review in 2007 concurred with these findings but noted that the supporting evidence was based mainly on one large study. This led to oral nimodipine becoming standard care for patients after aSAH ^83^. Interestingly, the review found no statistically significant results to support the use of other calcium antagonists, magnesium sulphate, or intravenous administration of nimodipine.

Magnesium sulphate is a non-competitive inhibitor of calcium channels and has vasodilatory and neuroprotective properties, similar to nimodipine. Hypomagnesaemia is common in patients after aSAH, appears to be proportional to the severity of the bleed, and is predictive of DCI ^84^. Magnesium sulphate has also been shown to reduce cerebral artery narrowing and the size of ischaemic lesions after aSAH in animal models ^85^. However, the Neurocritical Care Society guidelines advise against the routine administration of magnesium in patients with aSAH ^17^. This is supported by data from the intravenous magnesium sulphate for aneurysmal subarachnoid haemorrhage (IMASH) and MASH-2 trials and a recent meta-analysis demonstrating no beneficial effect of magnesium in this group of patients ^86– 88^. A *post hoc* analysis of the IMASH trial reported an association between high plasma levels of magnesium and worse clinical outcomes ^89^.

In summary, one calcium channel antagonist, nimodipine, has been shown to be effective in the prevention and treatment of DCI after aSAH whilst other calcium channel antagonists and a non-competitive inhibitor of calcium channels appear to have little effect on, or worsen outcomes.

It remains unclear how nimodipine exerts its neuroprotective effects but its action seems independent of any effect on large vessel narrowing ^90, 91^. It was thought that nimodipine may exert its effect by stopping calcium influx at a neuronal level, but no beneficial effect has been seen from administration in patients after ischaemic stroke or traumatic brain injury ^92– 94^. In addition to this, a recent systematic review found no benefit from nimodipine after traumatic SAH, suggesting that the mechanism of action of nimodipine is unique to aSAH ^95^. Nimodipine has two properties that it does not share with other calcium channel antagonists. Firstly, it increases endogenous fibrinolytic activity, which may reduce the incidence of microthrombosis ^96^. Secondly, it antagonises cortical spreading ischaemia in rats, which may be one of the culprits in DCI and is discussed in further detail below ^97^.

## Contemporary hypotheses

### Early brain injury

Early brain injury (EBI) refers to damage to the brain in the first 72 hours after the haemorrhage. There are a number of pathophysiological events in this time period that could influence later complications, such as DCI, and much of our understanding is derived from experimental data. One of these changes is a severe rise in intracranial pressure leading to decreased cerebral perfusion pressure, cessation of cerebral blood flow and ultimately global ischaemia and oedema ^98– 100^. The intracranial hypertension at ictus is often greater than systolic blood pressure, and the rate of increase and peak intracranial pressure appears to be proportional to the amount of arterial blood extravasating into the subarachnoid spaces from the aneurysm ^101– 103^. Cerebral spinal fluid outflow obstruction, in addition to hydrocephalus, further exacerbates intracranial hypertension ^104, 105^. However, the increase in intracranial pressure is not uniform and there are two distinct groups of patients in terms of their intracranial hypertension. The first, more common, scenario is an increase in intracranial pressure to the arterial diastolic pressure which then decreases to just above the patient’s baseline intracranial pressure ^102^. These patients typically have a small volume haemorrhage with cerebral oedema. The second type of increased intracranial pressure is sustained due to either a progressive haematoma or acute hydrocephalus ^104, 105^.

The cerebral oedema seen after aSAH is often present on admission CT scans and becomes more common, being present in up to 20% of patients by day 6 ^98^. Cerebral oedema is itself a poor prognostic factor after aSAH ^98, 106, 107^. The global cerebral ischaemia that occurs during the initial aSAH may lead to the disruption of the blood-brain barrier, and initiate cell death mechanisms and inflammatory responses which all contribute to cerebral oedema. Regulated and unregulated neuronal cell death appears to occur within 24 hours after aSAH and as early as 40 minutes after the initial injury ^108– 110^. Serum and cerebrospinal fluid (CSF) levels of pro-inflammatory cytokines and vasoactive factors, such as tumour necrosis factor-α, interleukin-6, and interleukin-1 receptor antagonist, correlate with DCI and poor outcomes ^111, 112^.

In addition to these inflammatory responses, blood degradation products are thought to contribute to DCI and perhaps removing blood from the subarachnoid space may improve outcomes ^30, 113^. Continuous cisternal drainage and intrathecal administration of thrombolytics have been trailed with reports of success, and results of the EARLYDRAIN trial comparing continuous lumbar-CSF drainage with standard treatment are awaited ^114, 115^. A meta-analysis of the use of intrathecal thrombolytics suggested a reduction in the incidence of DCI but these findings were not statistically significant after excluding one study, which included intrathecal nimodipine in addition to thrombolytic therapy ^116^.

Cerebral autoregulation, the ability of blood vessels to maintain constant cerebral blood flow (CBF) with arterial blood pressures between ~60 and 150 mmHg, is impaired after the aneurysm rupture ^117– 119^. Once impaired, autoregulation starts to rely on cerebral perfusion pressure and blood viscosity. Because of this, any change in intracranial pressure or systemic arterial pressure can potentially worsen oedema and ischaemia.

A limitation to many of these theories is that the majority of data comes from animal studies of experimental aSAH models. Some authors have questioned whether we can reliably translate data derived from this model to human studies ^120, 121^. We await the results of a systematic review and meta-analysis of intracranial *in vivo* animal studies of EBI and delayed cerebral arterial vessel narrowing after aSAH ^122^. The review aims to analyse aSAH models and define standard experimental parameters and endpoints for the study of EBI after aSAH and aSAH models of delayed cerebral arterial vessel narrowing.

### Cortical spreading depolarisation

Cortical spreading depolarisation (CSD), also termed cortical spreading depression, reflects a wave of depolarisation that spreads across grey matter at 2–5 mm/min. CSD is not a new theory, nor is it limited to aSAH, and has been implicated in brain injuries and migraine ^123^. It occurs when a cation influx across cellular membranes exceeds the Na ^+^ and Ca ^2+^ pump action and is followed by water and shrinkage of the extracellular space by ~70% causing depression of EEG (electroencephalography) activity ^124, 125^. Because the Na ^+^ and Ca ^2+^ pump is ATP-dependent, to counteract the passive influx of cations across the membrane energy consumption increases, which leads to increased regional blood flow requirements. When there is a dysfunction of the vasculature in the region, as occurs after aSAH, severe microvascular spasm can occur, rather than vasodilation, causing “cortical spreading ischaemia” ^125^. There is evidence that CSD occurs after the initial aneurysm rupture from both animal and human studies, and it is thought that after each depolarisation hypoperfusion of the cortex occurs due to vasoconstriction ^126^. Furthermore, up to 75% of all CSD episodes occur between days 5 and 7 after the aSAH, which matches DCI chronology ^127^. Another link between CSD and DCI comes from the CoOperative Study on Brain Injury Depolarisations (COSBID), which demonstrated that repeated CSD preceded DCI with little evidence of “vasospasm” on digital subtraction angiography (DSA), albeit in a small sample (thirteen patients) ^128^.

### Microthrombosis

Increased levels of procoagulants have been seen prior to DCI, specifically an increased von Willebrand factor 72 hours after aSAH and increased platelet-activating factors on day 4 ^129– 132^. Microthrombi have also been identified at the autopsy of patients after aSAH, suggesting that they are involved in aSAH pathology ^132^. The rate of rebleeding following aSAH has been significantly reduced following tranexamic acid administration. However, it may have led to an increased incidence of DCI separate from large vessel narrowing, possibly because the antifibrinolytic therapy caused microthrombosis and promoted DCI ^133– 136^. Unfortunately, the results of studies investigating antiplatelet agents in the treatment of microthrombosis after aSAH have been largely negative, including those investigating prophylactic low-molecular-weight heparin ^137, 138^.

## Therapies

### Intrathecal therapies

Intrathecal administration of nicardipine, a dihydropyridine calcium channel blocker, has been demonstrated in a number of clinical studies with varying results. Susuki *et al.* examined a series of 177 patients with Fisher grade III aSAH undergoing aneurysmal clipping and cisternal drainage within 48 hours of the aSAH ^139^. Patients received 4 mg intrathecal therapy nicardipine every 12 hours on days 3–14 postoperatively. Of these patients, 11.3% had radiographic evidence of vessel narrowing and 5.7% had clinical signs of DCI. The authors note a significant reduction in “vasospasm” but also recognise that 18.6% of patients required a shunt operation. Shibuya *et al.* demonstrated a decreased incidence of DCI and angiographic vessel constriction by 20 and 26% respectively after prophylactic administration of 2 mg intrathecal therapy nicardipine *via* a cisternal drain when compared with control patients ^140^. More recent trials also report positive findings, but are limited to cases of refractory “vasospasm” and have very small sample sizes ^141, 142^. However, nicardipine is associated with probable vasodilation-associated headaches, intracranial infections and hydrocephalus, and positive long-term outcomes from large RCTs are lacking. The NEWTON trial is a phase I/IIa multicentre RCT administering intrathecal nimodipine in patients with aSAH ^143^. The trial uses EG-1962, a sustained delivery system of nimodipine in microparticles. These will be injected into the ventricles through an external ventricular catheter in patients undergoing coiling or clipping of ruptured aneurysms. It is thought that systemic effects are less likely to occur as nimodipine concentrations are much lower in the plasma than CSF ^144^. We await the results of this trial and subsequent progressive trials with interest.

### Pleiotropic interventions

Statins have been investigated as a potential treatment for DCI due to their multiple effects, although a recent meta-analysis of the four single-centre RCTs demonstrated no benefit from statins after aSAH ^145^. Despite evidence that statins can reduce the duration of impaired autoregulation after aSAH, two more recent multicentre RCTs found no benefit from statin administration after aSAH ^146– 148^.

Another potential agent in the treatment of DCI is cilostazol, a phosphodiesterase 3 inhibitor and platelet aggregation inhibitor that affects smooth muscle cells. A meta-analysis of two RCTs and two quasi-RCTs demonstrated amelioration of cerebral vessel narrowing and a benefit on outcome at discharge, even after excluding the lower quality studies ^149– 151^. A subsequent trial has echoed these findings, but only one study has reported long-term outcomes and did not demonstrate improved outcomes with cilostazol ^151, 152^.

## Conclusion

In summary, cerebral vessel narrowing is consistently seen after aSAH, but its location and severity is not predictably linked to DCI. There is no conclusive evidence to support the treatment of vessel narrowing in the management of DCI, despite some studies reporting improved outcomes, specifically after more invasive techniques. Nimodipine is the only effective treatment for DCI but we still do not understand how nimodopine exerts its neuroprotective effect, although it does not seem to work by reversing cerebral artery narrowing, at least not in large vessels. It is possible that we are not detecting microvascular vasoconstriction or ischaemia on CT and TCD and so our understanding of the pathology is limited. Furthermore, improved recognition of DCI clinically, from imaging and/or biochemical markers would not only allow for quicker treatment but also better assessment of interventions. DCI almost certainly has a multifactorial aetiology and it may be that only by combining interventions will we see improved outcomes, but first we must understand the aetiology. Understanding how nimodipine, the only drug with proven efficacy, exerts its effect may be the key to creating new interventions with improved efficacy. There remains a large amount of work to be done in understanding DCI and investigating future potential treatments.
